# Hookworm exposure decreases human papillomavirus uptake and cervical cancer cell migration through systemic regulation of epithelial-mesenchymal transition marker expression

**DOI:** 10.1038/s41598-018-30058-9

**Published:** 2018-08-01

**Authors:** Brittany-Amber Jacobs, Alisha Chetty, William Gordon Charles Horsnell, Georgia Schäfer, Sharon Prince, Katherine Ann Smith

**Affiliations:** 1 Institute of Infectious Disease and Molecular Medicine, University of Cape Town, Cape Town, 7925 South Africa; 2 Institute of Microbiology and Infection, University of Birmingham, B15 2TT Birmingham, UK; 3 Le Studium Institute for Advanced Studies Laboratory of Molecular and Experimental Immunology and Neurogenetics, CNRS-University of Orleans, Rue Dupanloup, 45000 Orléans, France; 4 Department of Human Biology, University of Cape Town, Cape Town, 7925 South Africa; 5 School of Medicine, Cardiff University, Cardiff, CF14 4XN UK

## Abstract

Persistent infection with human papillomavirus (HPV) is responsible for nearly all new cervical cancer cases worldwide. In low- and middle-income countries (LMIC), infection with helminths has been linked to increased HPV prevalence. As the incidence of cervical cancer rises in helminth endemic regions, it is critical to understand the interaction between exposure to helminths and the progression of cervical cancer. Here we make use of several cervical cancer cell lines to demonstrate that exposure to antigens from the hookworm *N. brasiliensis* significantly reduces cervical cancer cell migration and global expression of vimentin and N-cadherin. Importantly, *N. brasiliensis* antigen significantly reduced expression of cell-surface vimentin, while decreasing HPV type 16 (HPV16) pseudovirion internalization. *In vivo* infection with *N. brasiliensis* significantly reduced vimentin expression within the female genital tract, confirming the relevance of these *in vitro* findings. Together, these findings demonstrate that infection with the hookworm-like parasite *N. brasiliensis* can systemically alter genital tract mesenchymal markers in a way that may impair cervical cancer cell progression. These findings reveal a possible late-stage treatment for reducing cervical cancer progression using helminth antigens.

## Introduction

Cervical cancer is the fourth most common cancer worldwide, causing an estimated 266,000 deaths in 2012. The major global burden of cervical cancer occurs in low-and middle-income countries (LMICs), which experienced almost 87% of disease-related deaths in this period. It is estimated that by 2035, the incidence of cervical cancer will increase by 58% (from 444,546 to 702,152 cases) in developing regions compared to 6% (from 83,078 to 88,041 cases) in high-income countries (HICs)^1^. Of significant interest is that nearly 100% of new cervical cancers are attributable to persistent infection with HPV^2^.

HPV prevalence and cervical cancer incidence varies significantly within the LMIC region. Recent epidemiological evidence suggests that shifts in the HPV immune response resulting from concurrent soil-transmitted helminth (STH) infection significantly increased HPV prevalence^3^. Helminth infections infect over a billion individuals in LMIC^4^ and result in the loss of 20 million disability-adjusted life years (DALY - the amount of years lost due to illnesses, disability or premature death)^5^. Amongst the leading STH infections are the hookworms, which cause more morbidity than most other human parasitic infections and infect more than 700 million people worldwide^6^.

Helminth infections are well documented as providing some benefit to the host, by limiting autoimmune and allergic symptoms in infected individuals^7–10^. However, helminth infections are also reported to dampen the efficacy of vaccination^11^ and impair immune responses to co-infection^12,13^. Helminth infections also influence the risk of cancer but this is understood in only a limited number of contexts. For example, *Schistosoma haematobium* is classified as a group 1 biological carcinogen and is a conclusive cause of bladder cancer^14^. Here, antigen from *Schistosoma haematobium* has been shown to induce urothelial dysplasia and inflammation, whereas the eggs cause squamous cell carcinoma of the urinary bladder^15^.

In addition to altering HPV prevalence^3^, infection with STH alters the replication of the oncogenic Kaposi’s sarcoma herpesvirus by driving reactivation from latency^16^. However, how STH infections may systemically influence cervical cancer cell progression is unknown. In this study, we used the rodent model of human hookworm *Nippostrongylus brasiliensis* to identify how STH may influence cancer cell biology. We show here that *N. brasiliensis* exposure significantly reduced cervical cancer cell migration and the uptake of HPV. These data demonstrate that helminth infection can systemically modify cancer cell progression by significantly altering epithelial-mesenchymal transition (EMT) marker expression.

## Results

### *N. brasiliensis* L3 antigen decreases HeLa migration *in vitro*

**Figure 1:**
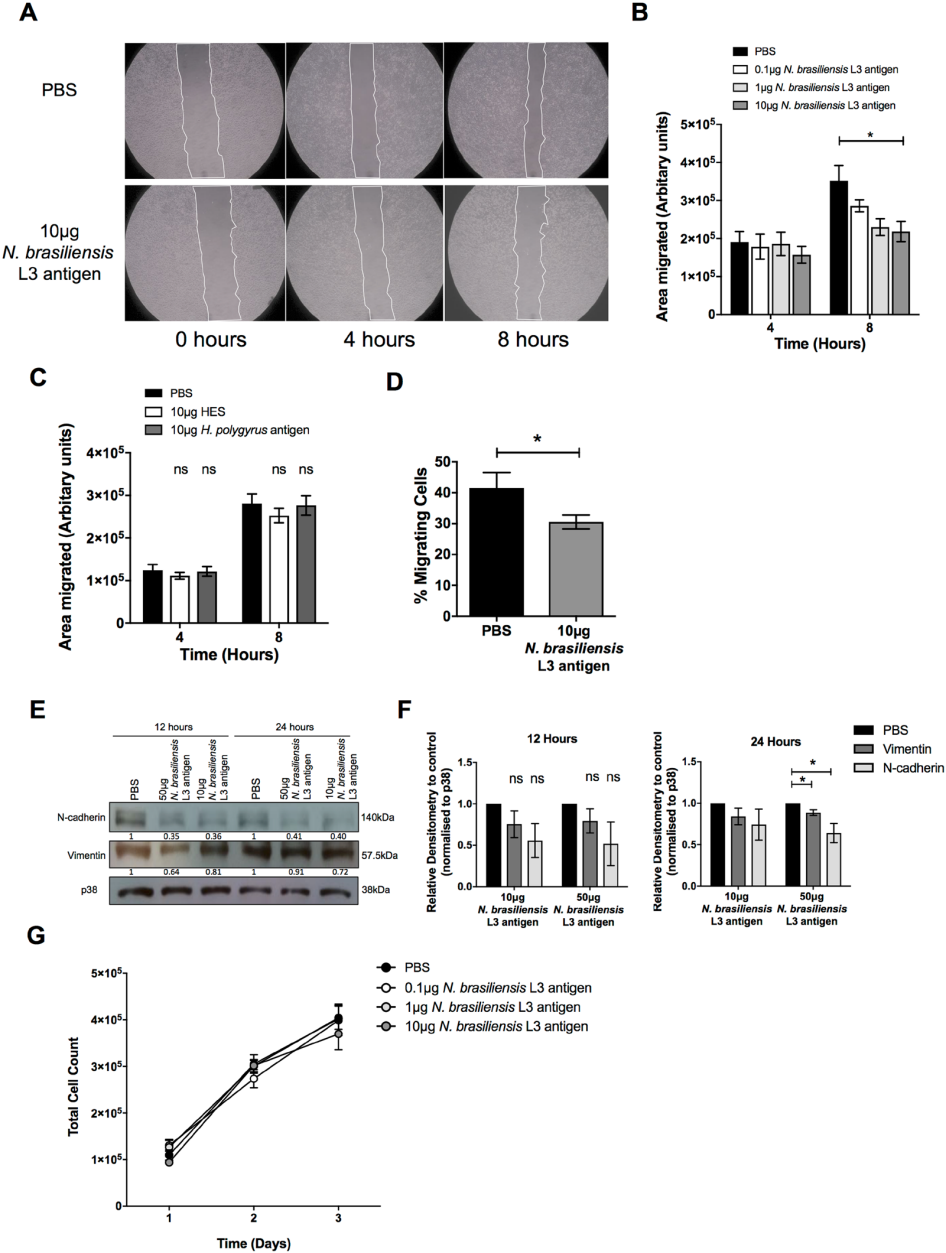
*N. brasiliensis* L3 antigen decreases HeLa migration and EMT marker expression. (**A**) Scratch motility assay showing HeLa cells exposed to 10 µg* N. brasiliensis* L3 antigen imaged at 4 and 8 hours post-wound formation and (**B**). Total area migrated calculated relative to wound area at time 0 hours for HeLa cells exposed to 0.1, 1 and 10 µg *N. brasiliensis* L3 antigen. (**C**) Scratch motility assay showing HeLa cells exposed to 10 µg *H. polygyrus* somatic antigen or 10 µg *H. polygyrus* excretory/secretory products (HES) imaged at 4 and 8 hours post-wound formation. (**D**) Transwell migration of HeLa cells determined at 24 hours following exposure to 10 µg* N. brasiliensis* L3 antigen or an untreated control. (**E**) Western blot analysis of N-cadherin, vimentin and p38 (loading control) was performed 12 or 24 hours following exposure of HeLa cells to 10 μg or 50 µg *N. brasiliensis* L3 antigen or an untreated control and (**F**). Densitometry readings were obtained using ImageJ in order to determine the changes in protein expression of N-cadherin and vimentin normalised to p38 and expressed as a fold-change relative to control wells. (**G**) Live cell counts over a 3-day period following exposure of HeLa cells to 0.1 µg, 1 µg and 10 µg *N. brasiliensis* L3 antigen or an untreated control. The figures shown are the result of (**G**). Two independent experiments, (**A–D** and **F**). Three independent experiments or (**E**). Representative of two independent experiments. The data was analysed using GraphPad Prism 6.01 and a parametric unpaired t-test was performed where ns = not significant, *p < 0.05 **p < 0.01 and ***p < 0.001. Error bars represent Standard Error of the Mean.

The impact of exposure to STH on cervical cancer cell proliferation and metastasis is unknown. Using an established *in vitro* two-dimensional scratch motility assay, we demonstrate that exposure of the HPV type 18 (HPV18) positive human cervical cell line HeLa to increasing concentrations of antigen isolated from the third-stage larvae (L3) of the hookworm *N. brasiliensis* L3 antigen for 8 hours, significantly decreased cervical cancer migration (Fig. 1A and B). Antigens from the solely gastrointestinal nematode *H. polygyrus* contain immune-modulatory components^17^ and were expected to impact HeLa migration. However, somatic antigen derived from the adult stage of *H. polygyrus* or the excretory/secretory products of this nematode had no effect on the migration of HeLa cells (Fig. 1C). Transwell migration assays also show that, compared to untreated cells, 10 μg *N. brasiliensis* L3 antigen significantly inhibited HeLa cell migration by 1.36 fold (Fig. 1D). To determine if the impact of *N. brasiliensis* L3 antigen on cervical cancer cell migration was associated with changes in markers of EMT, we assessed the expression of E-cadherin, N-cadherin and vimentin by western blotting. Although we could not detect E-cadherin in HeLa cells, we found that exposure of HeLa cells to 10 μg *N. brasiliensis* L3 antigen resulted in a trend toward reduced expression of N-cadherin and vimentin by 12 and 24 hours, compared to untreated cells. Treatment of HeLa cells to a high dose of 50 μg *N. brasiliensis* L3 antigen significantly reduced expression of N-cadherin and vimentin by 24 hours, compared to untreated cells (Fig. 1E and F). There was no change in the proliferative ability of HeLa cells exposed to increasing concentrations *N. brasiliensis* L3 antigen (Fig. 1G).

### *N. brasiliensis* L3 antigen decreases Ca Ski and C33-A migration *in vitro*

**Figure 2:**
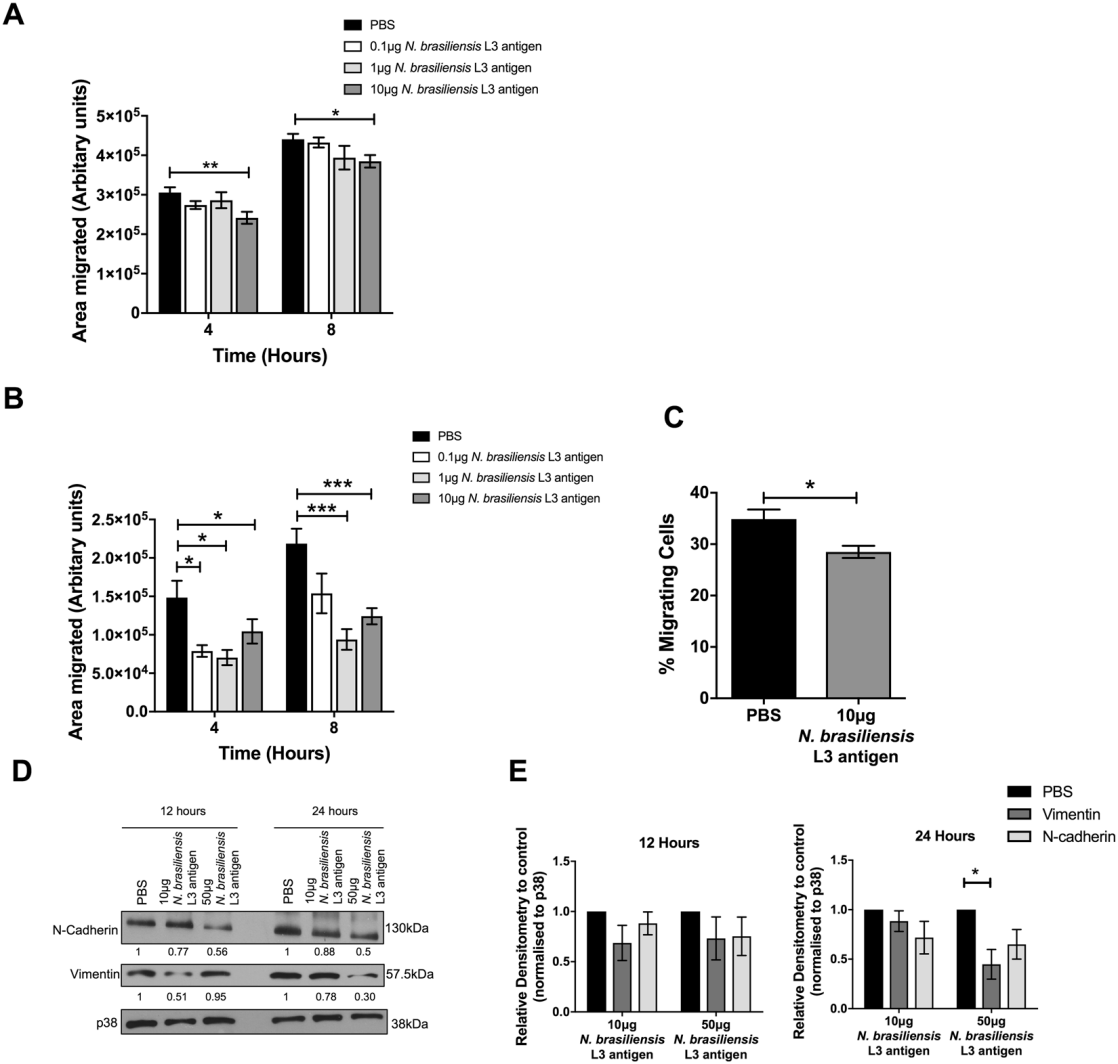
*N. brasiliensis* L3 antigen decreases HPV negative and positive cervical cancer cell migration. (**A**) Scratch motility assay showing Ca Ski cells exposed to 0.1 µg, 1 µg or 10 µg *N. brasiliensis* L3 antigen and imaged at 4 and 8 hours post-wound formation and the total area migrated calculated relative to wound area at time 0 hours. (**B**) Scratch motility assay showing C33-A cells exposed to 0.1 µg, 1 µg and 10 µg *N. brasiliensis* L3 antigen imaged at 4 and 8 hours post-wound formation. (**C**) Transwell migration of C33-A cells determined at 24 hours following exposure to 10 µg *N. brasiliensis* L3 antigen or an untreated control. (**D**) Western blot analysis of N-cadherin, vimentin and p38 (loading control) was performed 12 or 24 hours following exposure of C33-A cells to 10 μg or 50 µg *N. brasiliensis* L3 antigen or an untreated control and (**E**). Densitometry readings were obtained using ImageJ in order to determine the changes in protein expression of N-cadherin and vimentin normalised to p38 and expressed as a fold-change relative to control wells. The figures shown are the result of (**A**–**C**). Three independent experiments, (**E**). Two independent experiments, or (**D)**. Representative of two independent experiments. The data was analysed using GraphPad Prism 6.01 and a parametric unpaired t-test (**A**–**C**) or two-way ANOVA (**D**) was performed where ns = not significant, *p < 0.05 **p < 0.01 and ***p < 0.001. Error bars represent Standard Error of the Mean.

The impact of *N. brasiliensis* antigen on cervical cancer cell migration was validated in the HPV16 positive human cervical cancer cell line Ca Ski. Indeed, 10 μg of *N. brasiliensis* L3 antigen significantly decreased Ca Ski cell migration by 4 and 8 hours *in vitro* (Fig. 2A). To determine whether the HPV status of cervical cancer cells influences the effect of *N. brasiliensis* L3 antigen on cervical cancer cell migration we repeated the above experiments in the HPV negative human cervical cancer cell line C33-A. Our results show that 0.1 µg, 1 µg and 10 μg *N. brasiliensis* L3 antigen significantly reduced the migration of C33-A cells in scratch motility assays (Fig. 2B). The same results were obtained for 10 μg *N. brasiliensis* L3 antigen in transwell migration assays (Fig. 2C). Furthermore, both 10 μg and 50 μg *N. brasiliensis* L3 antigen reduced the expression of N-cadherin and vimentin in C33-A cells, compared to untreated cells (Fig. 2D and E).

### *N. brasiliensis* infected mice have reduced genital tract EMT marker expression

**Figure 3:**
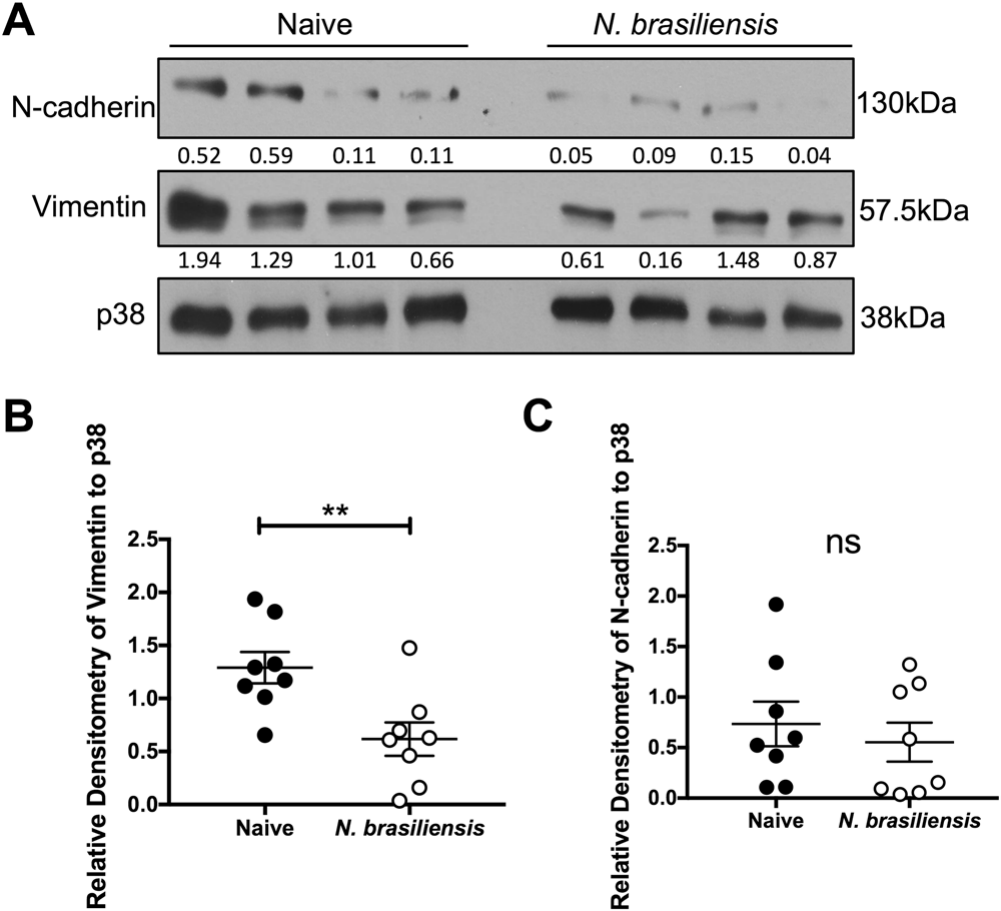
Exposure to *N. brasiliensis* decreases the expression of murine genital tract EMT markers. (**A**) Western blot analysis of N-cadherin, vimentin and p38 (loading control) within the female genital tract was performed 9 days following *N. brasiliensis* infection or in naïve BALB/c mice (**B**). Densitometry readings were obtained using ImageJ in order to determine the changes in protein expression of N-cadherin and vimentin as a ratio to p38 expression. The data shown is result of (**B** and **C**). Two independent experiments or (**A**). Representative of two independent experiments with n ≥ 4 mice per group. The data was analysed using GraphPad Prism 6.01 and a parametric unpaired t-test was performed where ns = not significant, *p < 0.05 **p < 0.01 and ***p < 0.001. Error bars represent Standard Error of the Mean.

While our data demonstrate that the expression of N-cadherin and vimentin is significantly reduced in HeLa and C33-A cervical cancer cell lines following exposure to *N. brasiliensis* L3 antigen *in vitro*, it is not known how *N. brasiliensis* infection impacts on the expression of these markers in non-cancerous tissue. We thus investigated this and demonstrate that infection significantly reduced the expression of vimentin in the female genital tract, while resulting in a trend toward decreased expression of N-cadherin, when compared to that of naïve mice (Fig. 3A–C). Indeed, the densitometric analyses of the western blots show that the average expression of vimentin and N-cadherin in infected mice are 0.62 and 0.55 compared to 1.29 and 0.73 in naïve mice respectively.

### *N. brasiliensis* L3 antigen decreases HeLa cell-surface vimentin expression

**Figure 4:**
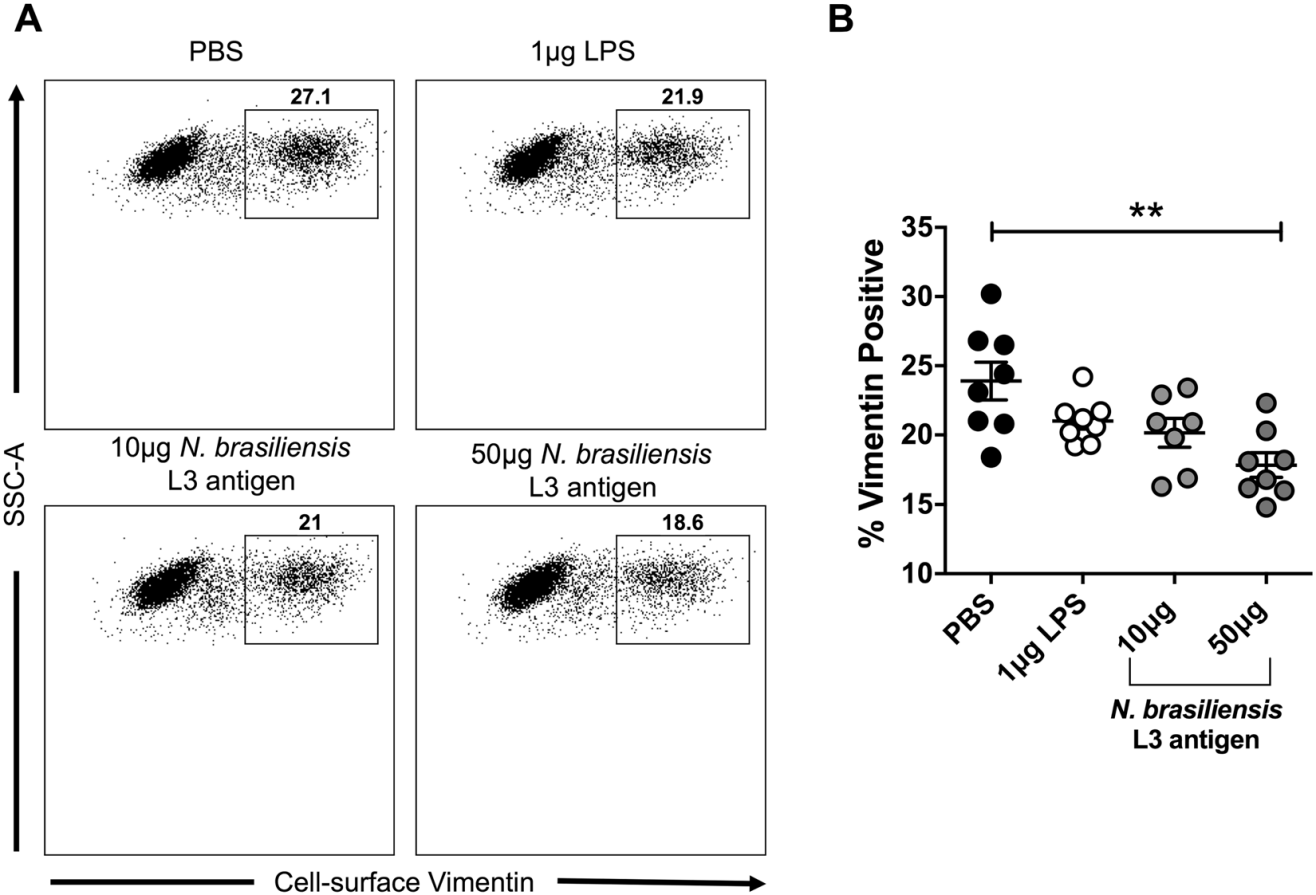
*N. brasiliensis* L3 antigen decreases HeLa cell-surface vimentin expression. (**A**) Representative flow cytometry plots and (**B**). Graphical representation of cell-surface vimentin expression by HeLa cells 12 hours following exposure to 10 µg or 50 µg *N. brasiliensis* L3 antigen, 1 µg LPS or an untreated control. The figure shown is a result of two independent experiments. The data was analysed using FlowJo Version 10 and GraphPad Prism 6.01 and a parametric unpaired t-test was performed where ns = not significant, *p < 0.05 **p < 0.01 and ***p < 0.001. Error bars represent Standard Error of the Mean.

Gastrointestinal helminths have been implicated in increasing the susceptibility of cervical tissue to the cancer-causing virus HPV^3^. Interestingly, cell-surface vimentin expression has been shown to act as a restriction factor on HeLa cells for HPV pseudovirions^18^. Although our western blot data demonstrate that *N. brasiliensis* L3 antigen significantly reduces the expression of vimentin in cervical cancer cells (Figs 1G and 2D), it is not known how antigen exposure alters cell-surface vimentin expression or the uptake of HPV pseudovirions. Using flow cytometry, we demonstrate that 12 hours post-treatment of HeLa cells with 50 µg of *N. brasiliensis* L3 antigen, the expression of cell-surface vimentin was significantly decreased when compared to untreated cells (Fig. 4A and B). When we screened the *N. brasiliensis* L3 antigen by *limulus* amebocyte lysate (LAL) assay we found it to contain low levels of endotoxin (2.80EU/ml) and we also demonstrate that endotoxin (from 1 µg of lipopolysaccharide) had no significant impact on the expression of cell-surface vimentin (Fig. 4B).

### *N. brasiliensis* L3 antigen decreases HPV16 pseudovirion internalisation in HeLa cells

**Figure 5:**
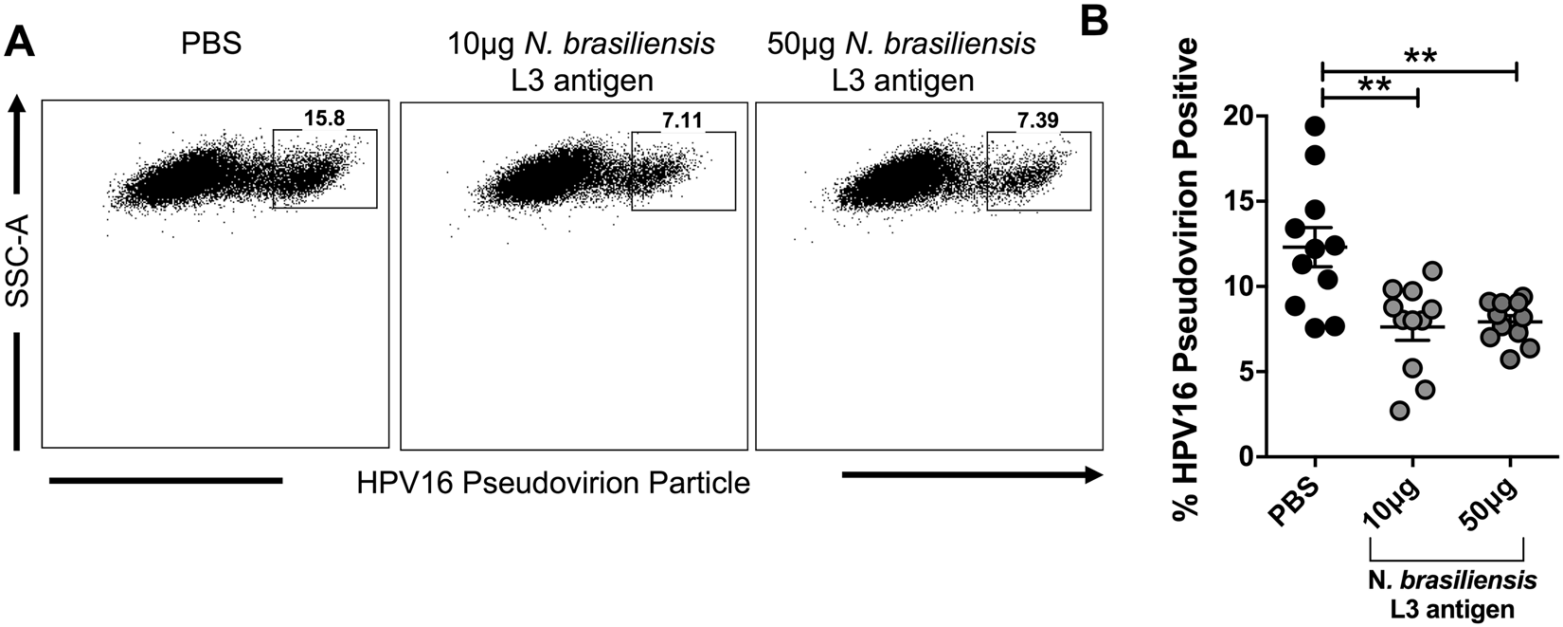
*N. brasiliensis* L3 antigen decreases HPV16 pseudovirion internalisation in HeLa cells. (**A**) Representative flow cytometry plots and (**B**). Graphical representation of Alexa-488 labelled HPV16 pseudovirion uptake by HeLa cells 12 hours following exposure to 10 µg or 50 µg *N. brasiliensis* L3 antigen or an untreated control. This figure is a result of three independent experiments. The data was analysed using FlowJo Version 10 and GraphPad Prism 6.01 and a parametric unpaired t-test was where ns = not significant, *p < 0.05 **p < 0.01 and ***p < 0.001. Error bars represent Standard Error of the Mean.

Due to the significant decrease in cell-surface vimentin expression observed following the exposure of HeLa cells to *N. brasiliensis* L3 antigen (Fig. 5), we assessed whether this may correlate with an expected increase in pseudovirion internalization. However, using flow cytometry, we demonstrate that 10 μg and 50 µg of *N. brasiliensis* L3 antigen significantly decreased the uptake of fluorescently labelled HPV16 pseudovirions by HeLa cells when compared to untreated cells (Fig. 5A and B).

## Discussion

Despite specific helminths being irrefutable causes of cancer, certain other helminths have been shown to modulate the host immune response by promoting immune-regulatory mechanisms^19^. While the exact mechanism that these helminths employ to achieve this is still emerging, the potential for their use in impeding cancer progression is worth thorough investigation. Epidemiological studies have revealed a positive correlation between STH infection and susceptibility to the oncogenic virus HPV^3^. However, it is currently unknown how exposure to STH helminths influences cervical cancer cell growth and metastasis. For these reasons, this study aimed to identify a possible mechanism by which helminths alter cervical cancer behaviour.

While *N. brasiliensis* L3 antigen had no significant effect on cervical cancer cell proliferation, high concentrations of the antigen significantly decreased the expression of EMT markers, N-cadherin and vimentin. Interestingly, the excretory/secretory products or somatic antigen from a solely gastrointestinal nematode *H. polygyrus* had no significant effect on the migration of HeLa cells, despite having a known immune-modulatory potential^17^. This suggests a selective inhibitory effect of hookworm antigen from *N. brasiliensis* on cervical cancer cell migration, which is likely to result from differences in the parasite lifecycle and the composition of their antigenic components. N-cadherin, a transmembrane cell adhesion molecule (NCBI Gene ID: 1000), and vimentin, an intermediate filament protein (NCBI Gene ID: 7431), are both expressed in mesenchymal cells and therefore, are used as markers of cells which are undergoing or have undergone EMT. Both N-cadherin and vimentin have been associated with the transition of epithelial cells to a more invasive and metastatic mesenchymal cell type^20^, which is associated with a poor outcome in cancer progression^21,22^. As a consequence of this decrease in EMT marker expression, a significant decrease in the migration of *N. brasiliensis* L3 antigen treated HeLa cells was observed in a two-dimensional scratch motility assay and a transwell migration assay. However, while the clinical relevance of this 1.36 fold decrease in migration requires further investigation, this result is comparable to what has previously been defined as a significant decrease in HeLa migration following drug treatment^23^. This observed decrease in EMT marker expression and migration was confirmed in another HPV positive cell line Ca Ski as well as a HPV negative cell line C33-A. Furthermore, the importance of this result was confirmed *in vivo* where genital tract tissue from *N. brasiliensis* infected mice displayed drastically reduced N-cadherin and vimentin expression compared to that of naïve mice.

As far as we are aware, this is the first study to reveal a role for hookworm exposure in reducing cervical cancer cell migration, through modulation of cell surface EMT markers. A report in 2005 described increased vacuolation and loss of cell contact by HeLa cells following exposure to the excretory/secretory products of the sheep nematode parasites *Haemonchus contortus* and *Ostertagia circumcincta*^24^. Interestingly, both products were reported to increase the permeability of Caco-2 cell monolayers, through re-distribution of the tight junction proteins zona occludens-1 and occludin^25^. We hypothesise that components of helminth antigens can alter the permeability of Caco-2 cells and the migration and invasion of cervical cancer cells through modulation of cell surface markers common to formation of tight junctions and the EMT process. More investigation is required to determine the precise mechanisms underlying how *N. brasiliensis* exposure significantly reduces EMT marker expression.

While an increase in cellular vimentin has been associated with a poor outcome in cancer progression^20^, cell-surface vimentin has been implicated as a restriction factor for HPV pseudovirion entry^18^. A recent epidemiological study by Gravitt *et al*., also revealed a positive correlation between STH infection and susceptibility to HPV^3^. For this reason, it was expected that the decrease in cell-surface vimentin expression observed when HeLa cells were exposed to *N. brasiliensis* L3 antigen would increase the susceptibility of HeLa cells to HPV16 pseudovirion internalisation. This was however, not the case and instead a significant decrease in pseudovirion internalisation was observed. Differing HPV subtypes have multiple receptors and restriction factors and therefore changes in the expression of only one of these proteins may not have an overall effect on virus internalization^26^. The sample size of the Gravitt *et al*., pilot study limited their ability to stratify HPV subtypes into their oncogenic potential (e.g. HPV16 and HPV18 strains), whereas stratification of parasitic species revealed high levels of *Ascaris lumbricoides*, *Trichuris trichiura*, *Giardia lamblia* and then *Ancylostoma duodenale* infection^3^. The protective effect of a hookworm (similar to *Ancylostoma duodenale* and *Necator americanus*) we note on HPV16 uptake may therefore have been missed. Interestingly, both this study and the Reese *et al*., paper^16^ implicate the t-helper type 2 cytokines (e.g. IL-4) associated with helminth infection in altering viral control. Further investigation is required in order to determine how *N. brasiliensis* modifies the expression of HPV receptors, including cell-surface vimentin and whether this is mediated systemically through type-2 responses.

The data presented here provides evidence that hookworm exposure could have a protective impact on cervical cancer cell progression, through inhibiting migration, EMT marker expression and HPV internalisation. Further in-depth analysis may reveal novel candidates for targeting and treating late-stage cancer.

## Methods

All methods were performed in accordance with The University of Cape Town’s guidelines and regulations.

### *N. brasiliensis* L3 antigen preparation

*N. brasiliensis* was maintained and used as described^27^. Briefly, *N. brasiliensis* L3 larvae were hatched and collected from the faecal material of Wistar rats, 5–7 days post-infection. Larvae were washed in fungizone before homogenization in Phosphate Buffered Saline (PBS) containing 1% protease inhibitor cocktail (Sigma). Following centrifugation, the supernatant was collected before quantification of protein by nanodrop.

### Cell culture

The human cervical epithelial cell lines, HeLa, Ca Ski and C33-A were purchased from the American Type Culture Collection (ATCC) and maintained in the appropriate specified media supplemented with 10% heat-inactivated fetal bovine serum (FBS), 100 µg penicillin and 100 µg streptomycin. Cells were maintained at 37 °C in an atmosphere of 5% CO_2_ and 95% humidity.

### Growth curve assay

The short-term growth of cells was monitored over a 3-day period as previously described^28^. 5 × 10^4^ cells were seeded per well, in triplicate, in a 24-well plate and *N. brasiliensis* L3 antigen (0.1 µg, 1 µg or 10 µg) or 1X PBS was added to each of the required wells. Live cells were counted 24, 48 and 72 hours post-treatment.

### Scratch motility assay

Cervical cancer cell migration was determined using a two-dimensional scratch motility assay^29^. 5 × 10^4^–2 × 10^5^ cells were seeded in triplicate, in a 24-well plate and allowed to reach confluency. Following serum starvation in medium containing 0% FBS for 24 hours, a sterile 2 µl pipette tip was used to make a vertical scratch in the cell monolayer of each well. Cells were treated with *N. brasiliensis* L3 antigen (0.1 µg, 1 µg or 10 µg), 10 µg *H. polygyrus* adult somatic antigen, 10 µg *H. polygyrus* excretory/secretory products (HES) or 1X PBS and subsequently viewed at 0, 4 and 8 hours post-wound formation. At each time point the total area of the scratch was imaged using ZoomBrowser Ex and pictures were taken using a non-phase contrast lens at 10X magnification (Canon PowerShot S50).

### Transwell migration assay

Cervical cancer cell migration was further characterized through an established transwell migration assay^29^, utilising 24-well hanging-inserts fitted with an 8 µm pore size membrane (Millicell Cell Culture Inserts Category No. MCEP24H48). 1 × 10^5^ serum starved cells were seeded in triplicate in media containing 1% FBS, onto the apical surface of each hanging-insert and placed into wells containing 10% FBS. Following the addition of *N. brasiliensis* L3 antigen (10 µg) or 1X PBS, the plates were incubated for 24 hours and the lower surface of the insert fixed with methanol and stained with crystal violet. Excess crystal violet was washed off the insert using distilled water before release of the crystal violet stain into 50% acetic acid. The absorbance of this solution was then quantified at 595 nm using a Rayto RT-2100C Microplate Reader.

### Western blotting

Western blotting was performed as previously described^30^. Primary antibodies used were as follows: mouse monoclonal anti-N-cadherin (Cell Signalling, #14215), rabbit polyclonal anti-vimentin (Cell Signalling, #3932) and rabbit polyclonal anti-p38 (Sigma-Aldrich, M0800). Signal was detected using peroxidase-conjugated goat anti-mouse or anti-rabbit antibodies (1:5000) and visualised by enhanced chemiluminescence (ECL) (Thermo Scientific, #34080 or Advansta, R-03031-D25). Densitometry readings were obtained using ImageJ in order to determine changes in protein expression.

### Western blotting on murine genital tract tissue

The female genital tract (excluding the ovaries) was removed from *N. brasiliensis* infected or naïve BALB/c mice. Prior to infection, female BALB/c mice were treated with 2 mg Medroxyprogesterone acetate (Depo Provera®) subcutaneously to synchronize estrous cycles. A week later, mice were infected subcutaneously with 500 L3 *N. brasiliensis* larvae and killed at day 9 post-infection. Tissue samples were sonicated in PBS containing 1% protease inhibitors before centrifugation. The protein concentration of the supernatant was determined and 20 µg of protein per sample was used to run a SDS-PAGE gel for western blot analysis.

### Flow cytometry on HeLa cell-surface vimentin expression

Flow cytometry was performed on 1 × 10^5^ cells treated with *N. brasiliensis* L3 antigen (10 µg or 50 µg), 1 µg lipopolysaccharide or 1X PBS in quadruplicate at 12 hours post-treatment^18^. Lidocaine-EDTA was used to lift the cells before staining with rabbit polyclonal vimentin H-84 antibody (Santa Cruz Biotechnology) and R-phycoerythrin-conjugated donkey anti-rabbit IgG (Jackson ImmunoResearch Laboratories, Inc), followed by acquisition on a BD LSR II Flow Cytometer and FlowJo, LCC software analysis.

### Flow cytometry on HPV16 pseudovirion internalisation

In order to determine the effect of *N. brasiliensis* L3 antigen on HPV internalisation, HeLa cells were exposed to fluorochrome labelled HPV16 pseudovirions and analysed by flow cytometry. HPV16 pseudovirion preparation, labeling, and quality controls were performed as previously described^18^. 1 × 10^5^ cells were seeded per well, in quadruplicate, in a 24-well plate and *N. brasiliensis* L3 antigen (50 µg or 10 µg) or 1X PBS added to each of the required wells for 12 hours. After this incubation period, 400 ng of Alexa-488 labelled HPV16 pseudovirions were added to each well (4 pg per cell) and incubated for 1 hour at 4 °C and then 30 minutes at 37 °C. Cells were then lifted using Trypsin-EDTA acquired on the BD LSR II Flow Cytometer and analysed using FlowJo, LCC software.

### Ethics

Section 20 dispensation to carry out animal research at the University of Cape Town was granted by the South African Department of Agriculture Fisheries and Food and by the UCT Health Sciences Animal Ethics Committee (Project number 014/027). All experimental protocols were approved by the University of Cape Town Animal Ethics Committee and were performed by researchers accredited by the South African Veterinary Council.

### Statistics

Data were assessed for normality and equal variance and were log transformed if required using the GraphPad Prism software (La Jolla, CA). For comparison between two groups, an unpaired T-test was used; where more than three groups were being tested, a parametric one-way analysis of variance with Tukey’s multiple comparison was used. Two-way ANOVA analysis was performed for groups split on two independent variables. NS on graphs denotes no statistical differences, *P < 0.05, **P < 0.01, and ***P < 0.001.

### Data availability

All data generated or analysed during this study are included in the published article.
